# Response to *Trypanosoma cruzi* by Human Blood Cells Enriched with Dentritic Cells Is Controlled by Cyclooxygenase-2 Pathway

**DOI:** 10.3389/fmicb.2017.02020

**Published:** 2017-10-25

**Authors:** Sandra C. H. Lonien, Aparecida D. Malvezi, Helena T. Suzukawa, Lucy M. Yamauchi, Sueli F. Yamada-Ogatta, Luiz V. Rizzo, Juliano Bordignon, Phileno Pinge-Filho

**Affiliations:** ^1^Laboratório de Imunopatologia Experimental, Departamento de Ciências Patológicas, Centro de Ciências Biológicas, Universidade Estadual de Londrina, Londrina, Brazil; ^2^Laboratório de Biologia Molecular de Microrganismos, Departamento de Microbiologia, Centro de Ciências Biológicas, Universidade Estadual de Londrina, Londrina, Brazil; ^3^Hospital Israelita Albert Einstein, São Paulo, Brazil; ^4^Laboratório de Virologia Molecular, Instituto Carlos Chagas/Fiocruz, Curitiba, Brazil

**Keywords:** *Trypanosoma cruzi*, cell invasion, human monocyte-derived dendritic cells, aspirin, celecoxib

## Abstract

Chagas disease (Cd) or American human trypanosomiasis is caused by *Trypanosoma cruzi* and affects ~7 million people, mostly in Latin America. The infective trypomastigote forms of the parasite can invade several human blood cell populations, including monocytes and dendritic cells (DC). Although these cells display a wide functional diversity, their interactions with *T. cruzi* via cyclooxygenase (COX) and cyclic adenosine monophosphate (cAMP) dependent pathways have not been analyzed. To exploiting this mechanism, DC-enriched peripheral human blood mononuclear cell populations (DC-PBMC) were used as our model. Our results showed that the treatment of these cell populations with celecoxib (CEL), a cyclooxygenase-2 selective inhibitor or SQ 22,536, an adenilate cyclase inhibitor, significantly caused marked inhibition of *T. cruzi* infection. In contrast, aspirin (ASA, a non-selective COX-1 and COX-2 inhibitor) treatment did not inhibit the infection of the cells by the parasite and was independent of nitric oxide (NO) production. The expression of co-stimulatory molecules CD80 and CD86 were similar on cells treated or not with both COX-inhibitors. The infection stimulated the release of TNF-α, IL-1β, IL-6, IL-8, and IL-10 production by infected cells. Treatment with ASA or CEL did not affect TNF-α, IL-6, IL-8, IL-10, and NO production by infected cells, but increased IL-1β production by them. Our results suggest a key role of COX-2 and cAMP pathways in *T. cruzi* invasion process of human blood cells and these pathways may represent targets of new therapeutic options for Cd.

## Introduction

The protozoan parasite *Trypanosoma cruzi* is the causative agent of Chagas disease (Cd) that is transmitted to humans from the feces of infected triatomine bugs. Nearly 7 million people worldwide are infected with *T. cruzi*, mainly in Latin America where the disease is endemic in many countries. However, Cd is becoming a global health concern and this can be partly attributed to population mobility between Latin America and the rest of the world. In fact, cases of Cd have already been detected in the United States, Canada, and in European and Western Pacific countries (Coura and Vinas, 2010; Bern, 2015; World Health Organization, 2017).

Cd is currently treated with either benznidazole or nifurtimox and these drugs have carcinogenic properties attributed to their active nitrofuran and nitroimidazole chemical groups, respectively (Wilkinson et al., 2011). This disease presents itself in two phases. An acute phase with nonspecific symptoms, which in most of the infected individuals is followed by an asymptomatic indeterminate chronic phase. About 5–40% of the cases develop a chronic symptomatic phase, after decades of acute infection, characterized by cardiac, digestive, or neurological conditions that may lead to death, with considerable psychological, social, and economic impacts (Machado et al., 2012; Dias et al., 2016).

Dendritic cells (DCs), macrophages, and natural killer (NK) cells respond to *T. cruzi* infection during the acute phase of Cd (Watanabe Costa et al., 2016). DCs modulate the immune response of the infected host and appear to depend on subtype and maturation level, influencing positively or negatively the development of the clinic forms of disease (Gil-Jaramillo et al., 2016).

The capacity of *T. cruzi* to infect and proliferate within human DC was revealed by Van Overtvelt et al. (1999), a biological event previously described for *Leishmania major* (Moll et al., 1995). *T. cruzi*-infected DCs reduces HLA-DR and CD40 expression and are not interleukin (IL)-12 and tumor necrosis factor (TNF)-α producer (Van Overtvelt et al., 1999). *T. cruzi* parasites also activates cord blood myeloid DCs, increasing the expression of CD40 and CD80 and promoting proliferation of CD8^+^ T cells and type-1-polarized response (Rodriguez et al., 2012a). It was also demonstrated that *T. cruzi* lysate elicits myeloid DCs independently of infection (Rodriguez et al., 2012b), a process recently revised by Gil-Jaramillo et al. (2016).

Microbe-induced microenvironments can influence DCs function also through indirect mechanisms, such as, inflammatory molecules or toxins (Palucka and Banchereau, 2002). For instance, prostaglandin E_2_ (PGE_2_) alters DCs function through PGE_2_ receptors and can modulate DCs to induce Th2 responses (Kalinski et al., 1999). In addition, it was demonstrated that the treatment with the analog of cyclic adenosine monophosphate (cAMP), dibutyryl cAMP, mimics the inhibitory effects induced by PGE_2_ in DCs (Harizi et al., 2003).

The interaction between human DCs and *T. cruzi* as well as yours specific functions are not fully understood and are only beginning to be unraveled (Gil-Jaramillo et al., 2016). It has not been analyzed how human cells interact with *T. cruzi* via a cyclooxygenase (COX) and cAMP dependent pathways.

In our work, we sought to test the effects of inhibition of cyclooxygenase-1 (COX-1) and 2 (COX-2) in DC-enriched peripheral human blood cell (DC-PBMC) populations infected with *T. cruzi* (Tc II genotype, Y strain). We show that parasite invade, survive and proliferate inside the DC-PBMCs. By comparing the different treatments with nonsteroidal anti-inflammatory drugs (NSAIDs), we demonstrate that inhibition of COX-2 and cAMP impair *T. cruzi* entry into DC-PBMCs and this is paralleled by higher IL-1β production by cells. Thus, COX-2 pathway and cAMP play an important role in the *T. cruzi* invasion process in human cells.

## Materials and methods

### Generation of DC-enriched peripheral human blood cell (DC-PBMC) populations

The study protocol was approved by the ethics committee of State University of Londrina (Process number: 5491/2012), National Committee for Ethics in Research (CONEP number: 5231). Blood samples (buffy coats) from healthy donors (*n* = 6) were collected at the University Hospital of State University of Londrina (UEL). All blood samples were collected in endotoxin-free heparinized tubes (BD Vacutainer® Sodium Heparin, 158 USP units) and processed within 1 h of collection.

Monocytes were isolated from PBMCs of healthy adult volunteers who were seronegative for Cd. Monocytes were isolated using lymphocyte separation medium (density 1.077 g/mL, Lonza, Walkersville, MD, USA) and gradient centrifugation at 750 × g for 20 min. Human monocytes were selected from mononuclear cells via adherence. The isolated monocytes (CD14^+^ PBMCs) were cultured for 6–7 days in RPMI 1640 (Lonza, Walkersville, MD, USA) medium containing 100 ng/mL IL-4 and 50 ng/mL GM-CSF, 10% inactivated fetal bovine serum (FBS), 100 UI/mL penicillin, 100 mg/mL streptomycin, and 2 mM L-glutamine (Gibco-BRL, Grand Island, NY), to be differentiated into immature monocyte-derived DCs (MoDCs) (Sallusto and Lanzavecchia, 1994; Figure S1 in Supplementary Material). The cells already showed CD11c^+^ expression and down-regulation of CD14 are consistent with DC phenotype, as assessed by flow cytometry using human mAb anti-CD11c (PE conjugated), human mAb anti-CD14 (FITC conjugated).

### Trypanosoma cruzi

*T. cruzi* (Tc II genotype, Y strain; Zingales et al., 2009) were maintained by weekly intraperitoneal inoculations to Swiss mice. Trypomastigote-infected blood (washed and diluted in PBS) was used to inoculate subconfluent cultures of Rhesus Monkey Kidney Epithelial Cells (LLC-MK2, ATCC CCL-7; American Type Culture Collection, Rockville, MD), as described previously (Malvezi et al., 2014b). Non-internalized parasites were removed after 24 h and cultures were maintained in RPMI 1640 medium (Lonza, Walkersville, MD, USA) containing 10% inactivated FBS (Gibco, BRL), 40 μg/mL gentamicin (Gibco, BRL), 100 units penicillin/mL (Gibco, BRL), 100 μg/mL streptomycin (Gibco, BRL). Free trypomastigote forms were detected in culture supernatants after 5 days post-infection.

### Treatment and viability of DC-PBMCs

Before *T. cruzi* infection, DC-PBMCs were incubated for 1 h at 37°C in a 5% CO_2_ atmosphere in the presence of different concentrations (0.312, 0.625, and 1.25 mM) of aspirin (ASA) or celecoxib (CEL) to evaluate the effects of the COX-inhibitors on trypomastigotes internalization (Malvezi et al., 2014a,b). The medium containing the inhibitors was removed and trypomastigotes were inoculated at a ratio of 5 parasites per cell. The system was incubated for 18–24 h at 37°C in a 5% CO_2_ atmosphere. Other treatments included incubation with PGE_2_ (1 or 10 μM; Lopez-Munoz et al., 2010), 20 μM SQ 22,536 (Procopio et al., 1999), or 10 μM Forskolin (Malvezi et al., 2014b) for 30 min at 37°C in a 5% CO_2_ atmosphere. One set of culture plates was used to quantify cytokines and nitric oxide (NO). Expression levels of co-stimulatory molecules CD80 and CD86 were similar on untreated and treated DC-PBMC, and suggests that treatment with ASA or CEL was not provoked cell activation (Figure S2 in Supplementary Material).

The viability of the untreated and treated DC-PBMCs was evaluated by using the dimethylthiazol diphenyl tetrazolium bromide (MTT, Sigma Chemical Co., USA) method, showing mitochondrial activity of viable cells. Cells were incubated with MTT (final concentration 0.5 mg/ml, 0.1%) at 37°C for 4 h. Supernatant was aspirated off and dimethyl sulfoxide (DMSO) was added to solubilize the formazan crystals. The supernatants were transferred to a new plates and absorbance (A) was read using a Synergy HTX multiplate reader (Biotek, USA), at a test wavelength of 570 nm and reference wavelength of 630 nm. Percentage of cell viability was calculated using the formula: % cell viability = (mean A in test wells)/mean A in control wells) × 100.

### Infection of DC-PBMC by *T. cruzi*

After the different treatments, DC-PBMCs were washed three times with PBS, fixed with methanol, stained with Giemsa (Merck), and observed with a light microscope. The percentage of infected DC-PBMCs and the mean number of amastigotes per infected cell were recorded by direct counting of at least 200 cells after microscopic examination (Van Overtvelt et al., 1999). For determination of levels of cytokines and NO analyses, cells were not fixed but were processed accordingly.

### Flow cytometry

FITC-labeled anti-CD80, PE-labeled anti-CD86 antibodies were obtained from BD Pharmingen (San Diego, CA, USA). Samples were analyzed using BD Accuri™ C5 Cytometer (Becton, Dickinson and Company, San Jose, CA, USA) and the data were analyzed using FlowJo version 10.0 (Tree Star Inc., Ashland, OR, USA). Inflammatory cytokines (IL-8, IL-1β, IL-6, IL-10, TNF-α, and IL-12p70 protein levels in a single sample) were determined in cell-free supernatants with the BD™ CBA Human Inflammatory Cytokines Kit (Becton, Dickinson and Company).

### Nitric oxide (NO) measurement

NO production was detected by measuring NO-derived nitrite accumulation level in the culture supernatants using Griess reagent (Sigma-Aldrich). Following 24 h-treatment of DC-PBMCs with COX-inhibitors [ASA or celecoxib (1.25 mM)] or SQ 22536 (20 μM), culture supernatants were transferred to a new 96-well microtiter plate and mixed with equal volume of Griess reagent. The plates were incubated at room temperature for 10 min and absorbance was determined at 540 nm. Nitrite concentrations were calculated using standard curve for sodium nitrite.

### Statistical analysis

Data were analyzed by parametric (one-way ANOVA with Tukey's post-test) and non-parametric (Kruskal–Wallis test, Dunn's post-test) statistical tests using Prism (version 5.0, GraphPad Software Inc., San Diego, CA, USA). Values are presented as mean ± SEM. Results were considered significant when *P* < 0.05.

## Results

### Inhibition of ciclooxigenase-2 impairs *T. cruzi* entry into DC-PBMC

**Figure 1:**
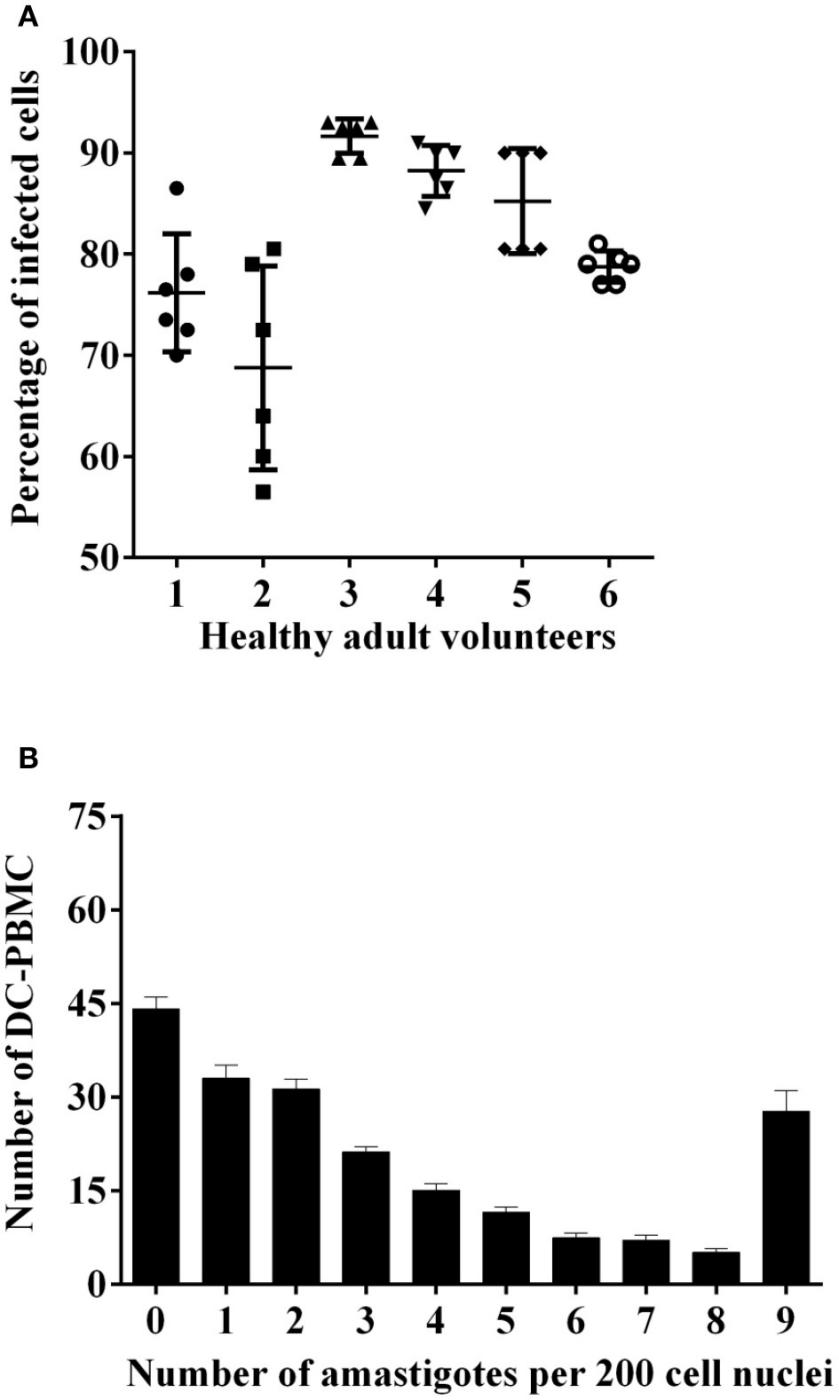
*T. cruzi*-infected DC-PBMC. DC-PBMC presented different susceptibilities to *T. cruzi* infection. DC-PBMC were incubated with *T. cruzi* trypomastigotes at parasite-to-cell ratio (5:1). After 18 h, the cultures were washed to remove free parasites and DC-PBMC were fixed with methanol and stained with Giemsa stain. Percentages of infected DC-PBMC **(A)** and mean numbers of amastigotes per infected DC-PBMC **(B)** were recorded after microscopy examination of at least 200 cells. Results are the mean ± standard error for six independent experiments with six different blood donors.

In a first set of experiments, we analyzed the infection rate of DC-PBMCs incubated with *T. cruzi* trypomastigotes at parasite-to-cell ratio 5:1 for 18 h (Van Overtvelt et al., 1999). DC-PBMCs presented different susceptibilities to parasite infection, the percentage of infected cells ranged between 68.75% (volunteer two) and 91.66% (volunteer tree), Figure 1A, with a mean of 80.64%. The mean number of amastigotes per infected DC-PBMCs ranged in the 18 h of culture (Figure 1B). For example, ~33 infected cells presented one amastigote/cell and ~28 infected cells presented nine amastigotes/cell recorded after microscopy examination of 200 cells (Figure 1B). These data indicated that *T. cruzi* invade, survive, and proliferate inside the DC-PBMCs.

**Figure 2:**
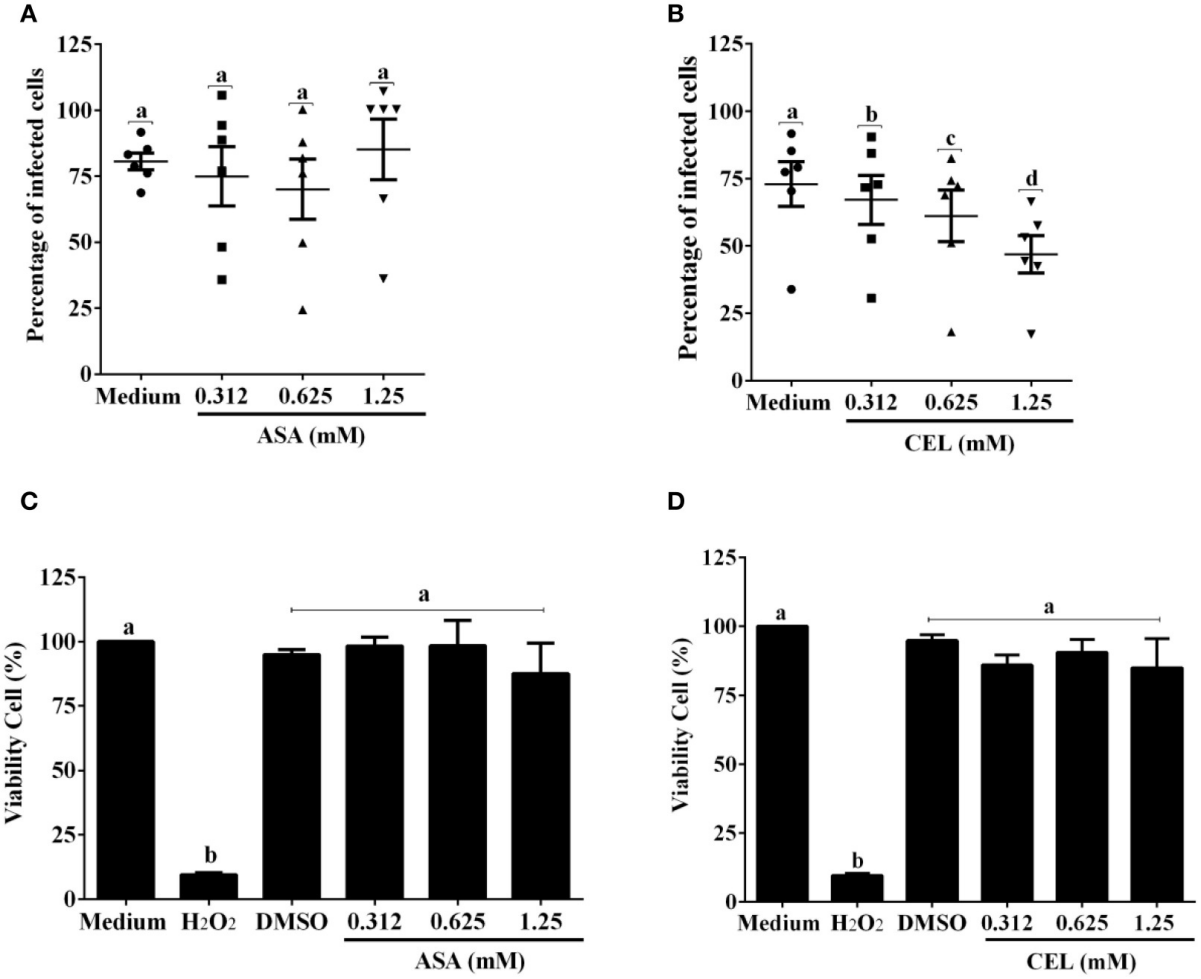
Inhibition of ciclooxigenase-2 impairs *T. cruzi* entry into DC-PBMC Percentage of infected cells from the interaction process between DC-PBMC treated for 1 hour with ASA **(A)** or CEL **(B)** [0.312, 0.625, and 1.25 μM] and exposed to 5:1 trypomastigotes of *T. cruzi* (Y strain) during 18 h. After DC-PBMC were fixed with methanol and stained with Giemsa stain. Quantification was carried out under a light microscope where the number of intracellular parasites was counted in a total of least 200 cells. MTT assay to measure cell viability in DC-PBMC after treatment with ASA **(C)** or CEL **(D)**. H_2_O_2_ (1,000 μM) was used as positive control. Values are the mean ± standard error of two experiments. DMSO, Dimethyl sulfoxide 0.05%. Results are the mean ± standard error for duplicate determinations, with six blood donors in each experiment. Means not sharing letter are significantly different (*P* < 0.05, one-way ANOVA with Tukey's post-test).

**Figure 3:**
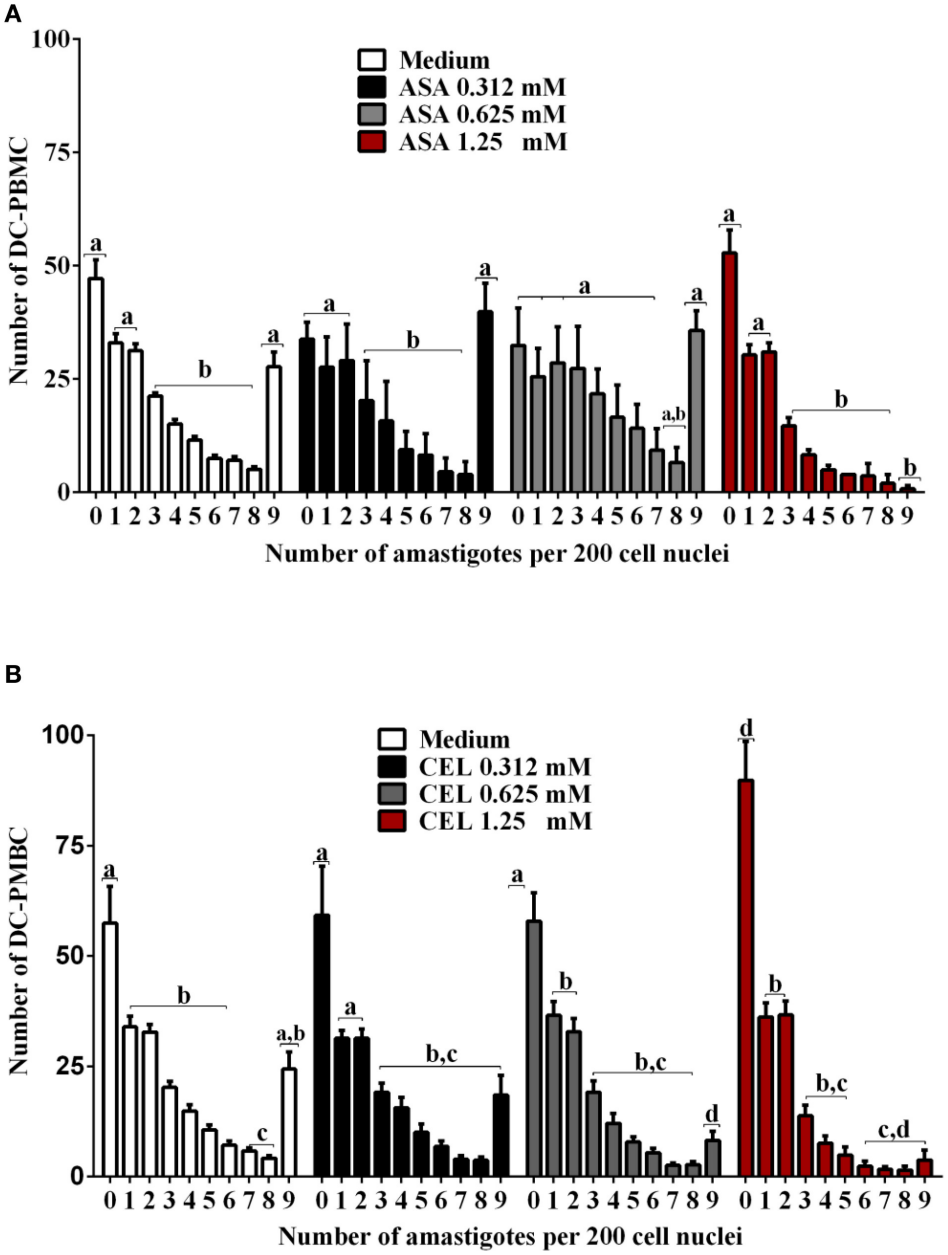
*Trypanosoma cruzi* infection levels of DC-PBMC. Number of infected DC-PBMC with nine amastigotes per cell decreased with the treatment for both COX-inhibitors used. Only 1.25 mM of ASA was able of provoked this reduction **(A)**. High (1.25 mM) and low concentrations of CEL (0.625 mM) provoked a decrease in the number of cells containing nine amastigotes per cell **(B)**. Results are the mean ± standard error for duplicate determinations, with three blood donors in each experiment. Means not sharing letter are significantly different (*P* < 0.05, one-way ANOVA with Tukey's post-test).

**Figure 4:**
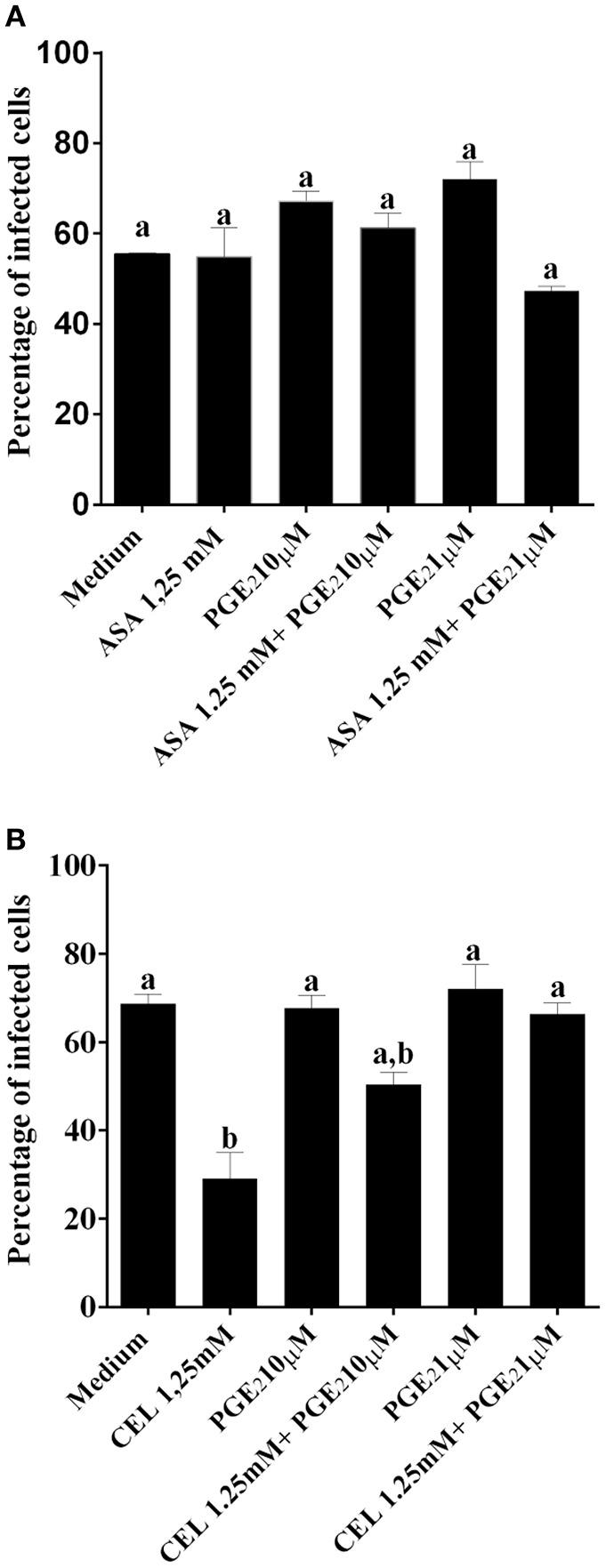
PGE_2_ restored the invasiveness of *T. cruzi* in DC-PBMC previously treated with CEL. DC-PBMC were treated for 30 min with separately either with or without PGE_2_ (1 or 10 μM) alone or in combination with ASA **(A)** or with CEL **(B)** and exposed to 5:1 trypomastigotes of *T. cruzi* (Y strain) during 18 h. After cells were fixed with methanol and stained with Giemsa stain. Results are the mean ± standard error for duplicate determinations, with three blood donors in each experiment. Means not sharing letter are significantly different (*P* < 0.05, one-way ANOVA with Tukey's post-test).

We next investigated whether the inhibition of COX-pathway with ASA (Figure 2A) or celecoxib (Figure 2B) would affect the infection rate of cells. First, we observed that treatments with COX-inhibitors did not interfere in DC-PBMC viability that was over 95% when analyzed by MTT reduction assay (Figures 2C,D). Only celecoxib markedly inhibited the *T. cruzi* entry into DC-PBMCs (Figure 2B). Interestingly, the mean number of infected DC-PBMC with nine amastigotes per cell decreased with the treatment for both inhibitors used (Figure 3). We observed that only 1.25 mM of ASA was able of provoked this reduction (Figure 3A). In contrast, both high (1.25 mM) and low concentrations of CEL (0.625 mM) provoked a decrease in the number of cells containing nine amastigotes per cell (Figure 3B). When PGE_2_ (1 or 10 μM) was added alone or in combination with ASA or CEL (Figure 4), the effect of CEL was inhibited, indicating an involvement of PGE_2_ in the internalization of *T. cruzi* trypomastigotes into DC-PBMC. Taken together, these data indicated that PGE_2_ synthesis inhibition using celecoxib (CEL) could improve human cells response against *T. cruzi* infection.

### Modulation of adenylyl cyclase activity regulates DC-PBMC invasion by *T. cruzi*

**Figure 5:**
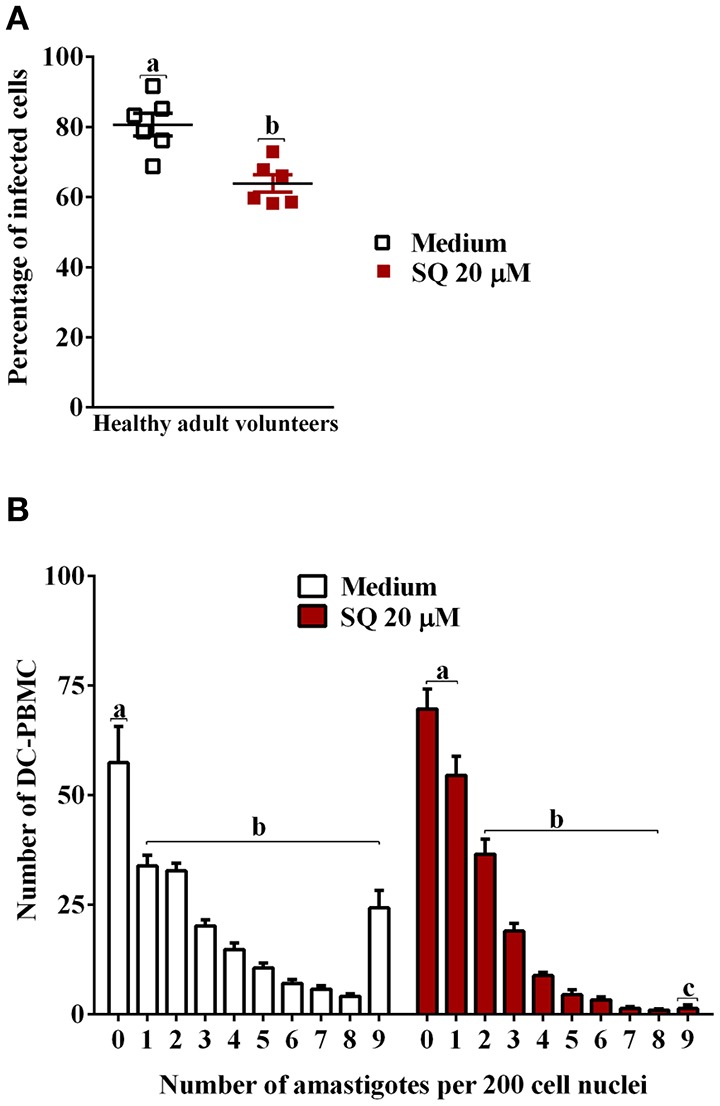
Adenylate-cyclase activity regulates the entry of *T. cruzi* into DC-PBMC. DC-PBMC were treated for 30 min separately either with or without SQ 22536 (20 μM) and exposed to 5:1 trypomastigotes of *T. cruzi* (Y strain) during 18 h. Inhibition of adenylate-cyclase decreases *T. cruzi* infection **(A)**. Number of infected DC-PBMC with nine amastigotes per cell decreased with the treatment **(B)**. Results are the mean ± standard error for duplicate determinations, with five **(A)** and six **(B)** blood donors in each experiment. Means not sharing letter are significantly different (*P* < 0.05, one-way ANOVA with Tukey's post-test).

**Figure 6:**
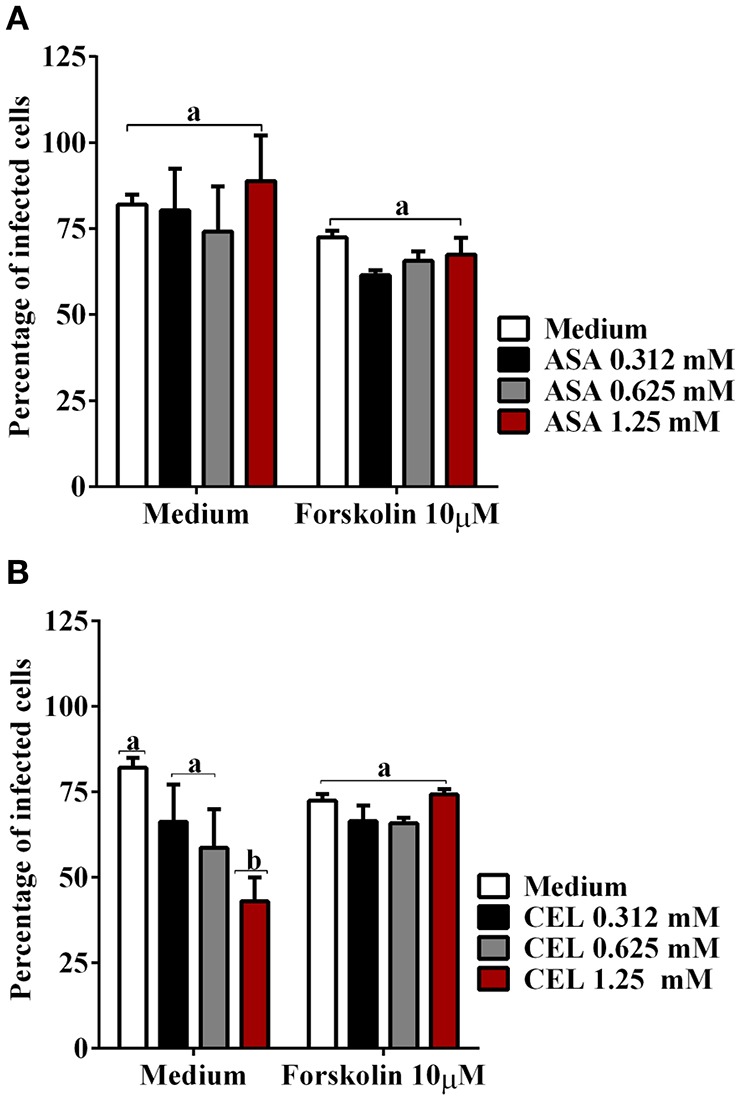
The activation of adenylyl cyclase with forskolin reverted CEL effects on *T. cruzi* entry. DC-PBMC were treated for 30 min with forskolin (10 μM) alone or in combination with ASA **(A)** or with CEL **(B)** and exposed to 5:1 trypomastigotes of *T. cruzi* (Y strain) during 18 h. The activation of adenylyl cyclase with forskolin did not affect cell invasion by trypomastigotes from Y strain (*P* > 0.05) **(A,B)**, but reverted CEL effects (*P* < 0.05) **(B)**. Results are the mean ± standard error for duplicate determinations, with three blood donors in each experiment. Means not sharing letter are significantly different (*P* < 0.05, one-way ANOVA with Tukey's post-test).

In the context of production of endogenous mediators, the immunomodulatory effects of PGE_2_ are largely derived from the ability to increase intracellular cAMP levels. We further examined the effect of adenylate-cyclase inhibition by SQ 22536 (20 μM) on the entry of *T. cruzi* into DC-PBMC. Treatment with this inhibitor reduced *T. cruzi* entry into human DCs (Figure 5A). The mean number of infected DC-PBMC with nine amastigotes per cell decreased with the SQ 22536 treatment (Figure 5B). SQ 22536 did not show cytotoxicity against uninfected DC-PBMC (data not shown). In addition, the activation of adenylyl cyclase induced by forskolin did not affect ASA treated-DC-PBMCs invasion by trypomastigotes from Y strain (*P* > 0.05, Figure 6A), but reverted CEL effects (Figure 6B, *P* < 0.05).

### Effects of ASA and CEL on innate inflammatory response of DC-PBMC cells infected with *T. cruzi*

**Figure 7:**
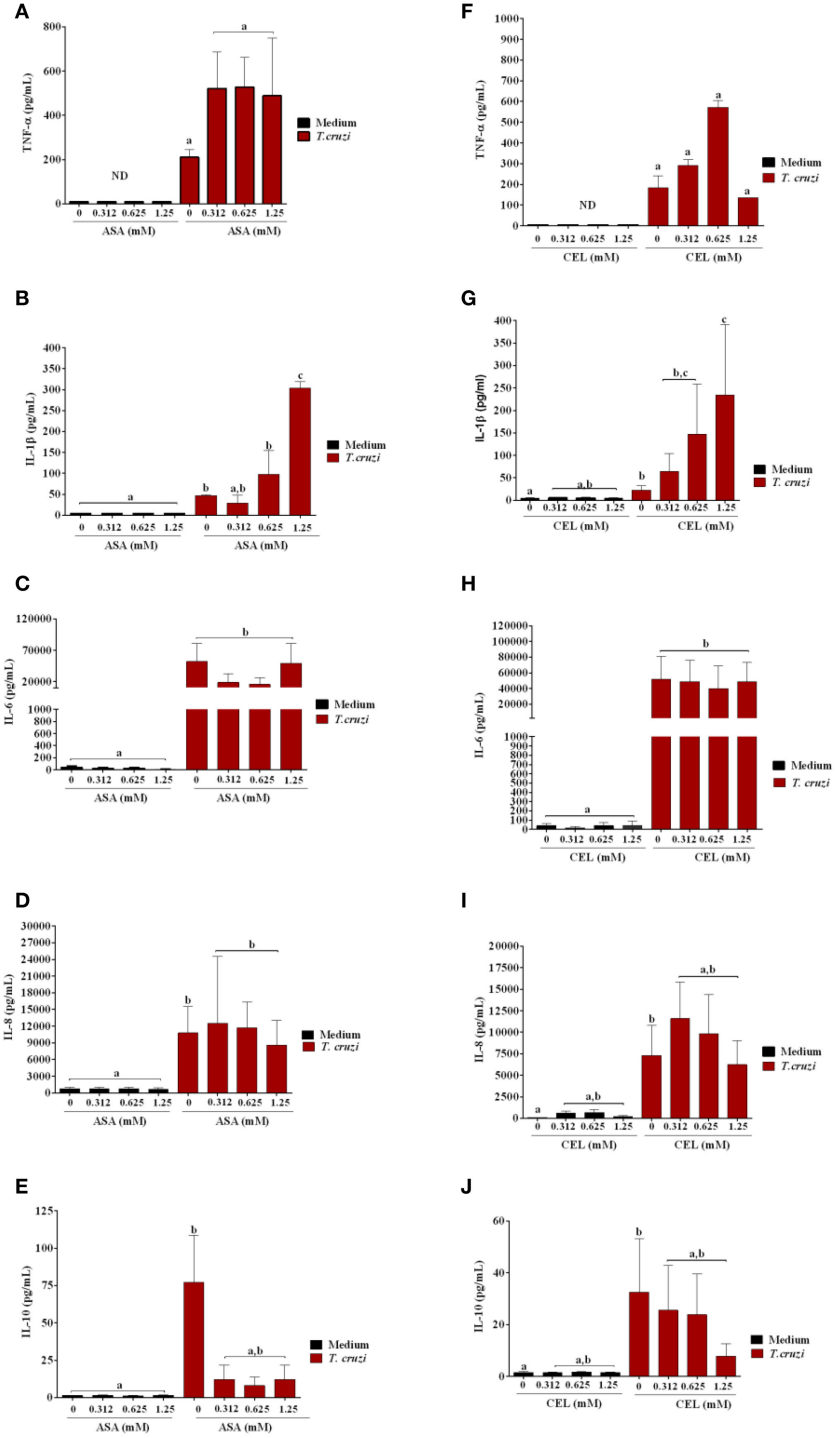
Effects of ASA and CEL on innate inflammatory response of DC-PBMC cells infected with *T. cruzi*. The levels of IL-8, IL-1β, IL-6, IL-10, and TNF-α were measured following a 24-h treatment of DC-PBMC infected or not with *T. cruzi*. Infection induced cytokine production in DC-PBMC **(B–J)**. The treatment with ASA or CEL did not affect TNF-α, IL-6, IL-8, IL-10 production by DC-PBMC **(A,C–J)**, but increased IL β production by them **(B,G)**. Representative results from at least two independent experiments are shown, with three blood donors in each experiment. Means not sharing letter are significantly different (*P* < 0.05, non-parametric, Kruskal-Wallis test, Dunn's post-test).

**Figure 8:**
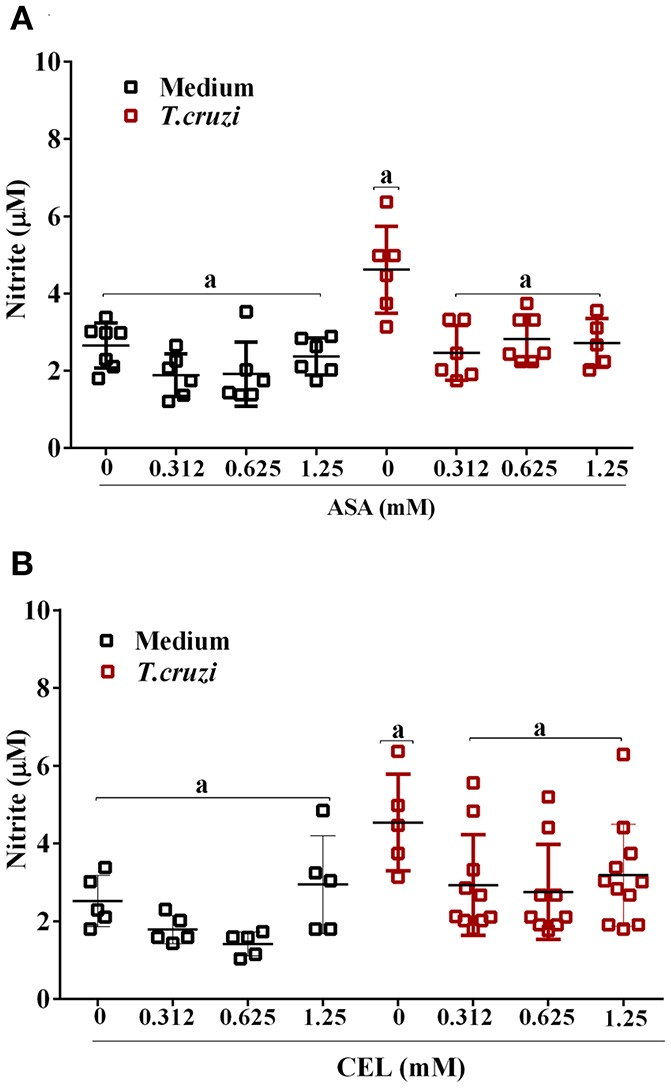
Nitric oxide (NO) production by DC-PBMC infected with *T. cruzi*. DC-PBMC were treated for 1 h with ASA **(A)** or CEL **(B)**. After treatment, the cells were washed and incubated with 5:1 trypomastigotes for 24 h. Nitrite levels in the supernatant were measured by Griess reaction. Results are the mean ± standard error of duplicate determinations. Three independent experiments were performed, with six individuals in each experiment. Means not sharing letter are significantly different (*P* < 0.05, one-way ANOVA with Tukey's post-test).

We analyzed the effects of ASA and CEL on cytokines and NO production by infected DC-PBMCs. The entry of parasite into cells stimulated TNF-α, IL-1β, IL-6, IL-8, IL-10 releasing (Figure 7). Treatment with ASA or CEL did not affect TNF-α, IL-6, IL-8, IL-10 (Figure 7) neither NO production by DC-PBMCs (Figure 8), but increased IL β production by them (Figures 7B,G). Levels of TNF-α were below the limit of detection of the assays in the absence of trypomastigotes (Figures 7A,F). Levels of IL-12 were also below the limit of detection in the presence and absence of trypomastigotes (data not shown).

## Discussion

Recent evidence suggests that temporary inhibition of COX-2 activity can facilitate parasite survival in the early stage of interaction of *T. cruzi* with host cells (Moraes et al., 2015). In *T. cruzi*-infected cells, an inflammatory response is activated, in which COX-2 catalyzes rate-limiting steps in the arachidonic acid pathway (Moraes et al., 2015). However, the involvement of COX-mediated prostaglandin (PG) production in the entry of *T. cruzi* into human DCs is unknown.

PG can up-regulate the expression of NO synthase, stimulating NO production by macrophages during *T. cruzi* infection, leading to a highly oxidizing environment capable of killing the parasite (Pinge-Filho et al., 1999; Durand et al., 2009; Ganzinelli et al., 2009). In fact, there are at least two mechanisms of oxidative stress generation, which can be dependent or independent of the balance of NO and PG production, and each mechanism being predominant according to the type of cell or mouse (Silva et al., 2003; Hideko Tatakihara et al., 2008; Ganzinelli et al., 2009). In addition, it was showed that phagocytosis of apoptotic T lymphocytes or neutrophils inhibits the parasite growth in macrophages in a manner dependent of TGF-β and PG. Hence, the NO content in macrophages decreases, the parasite proliferates (Freire-de-Lima et al., 2000; Lopes and DosReis, 2000; DosReis and Lopes, 2009; Maya et al., 2010). On the other hand, the inhibition of COX by indomethacin resulted in marked reduction of PGE_2_ in macrophages (Abdalla et al., 2008) and spleen cells from *T. cruzi*-infected mice (Pinge-Filho et al., 1999). Interestingly, in the presence of *T. cruzi*, COX-2 enzyme exhibited an oscillatory activity pattern in H9c2cells (an embryonic rat ventricular cell line) during the first 48 h post-infection, which correlated with the control of the pro-inflammatory environment in infected cells (Moraes et al., 2015).

Recent evidence suggests a role of COX-2 and PGE_2_ signaling through EP-2 receptor in the development of myocarditis during acute *T. cruzi* infection in mice. In fact, it was showed a marked reduction in the cardiac inflammatory infiltration in knockout mice deficient in the expression of COX-2 (COX-2^−/−^) and the prostaglandin PGE_2_ receptor EP-2 (EP-2^−/−^) infected with *T. cruzi* compared to infected wild type animals (Guerrero et al., 2015).

The role of COX during *T. cruzi* infection in mouse has been studied using non-selective inhibitors of COX-1 and COX-2, as well as COX-2-selective inhibitors (NSAIDs), with controversial results (Machado et al., 2011). The use of ASA (weakly more selective for COX-1 than COX-2), CEL (COX-2-specific inhibitor) and indomethacin (non-selective inhibitor of COX-1 and COX-2) increases mortality and parasitemia (in peripheral blood and cardiac tissue), regardless of the mouse model or *T. cruzi* strain used (Celentano et al., 1995; Hideko Tatakihara et al., 2008). Moreover, administration of NSAIDs may enhance mortality in mice infected with non-lethal *T. cruzi* strain (Sterin-Borda et al., 1996), but other studies showed that inhibition of COX-2 activity decreases the level of parasitism (Freire-de-Lima et al., 2000). In addition, beneficial and adverse effects of COX inhibitors have been reported, depending on the phase of *T. cruzi* infection and mouse strain (Machado et al., 2000). More recently, it was showed that NSAIDs modulate innate inflammatory response and inhibit the entry of *T. cruzi* into phagocytic cells (Lopez-Munoz et al., 2010; Molina-Berrios et al., 2013; Malvezi et al., 2014a) and non-phagocytic cells (Malvezi et al., 2014b) thereby controlling Cd progression (Machado et al., 2011; Mukherjee et al., 2011; Molina-Berrios et al., 2013).

Our data clearly show that the treatment of DC-PBMCs with CEL significantly inhibits internalization of trypomastigote forms of *T*. cruzi, and this event may be related with the inhibition of PGE_2_ synthesis. Our observations on DC-PBMCs infection corroborate with those obtained in experiments with human and murine cells infected by *T. cruzi* (de Araujo-Jorge, 1989; Van Overtvelt et al., 1999). These results strongly support that COX- pathway plays a fundamental role in parasite invasion of host cells (Lopez-Munoz et al., 2010; Malvezi et al., 2014a,b).

Invasion by *T. cruzi* may trigger cAMP releasing in host cells (Rodriguez et al., 1999; Caler et al., 2000). Here, we observed that the treatment of DC-PBMCs with SQ 22536 (adenylate-cyclase inhibitor) reduced parasite entry into these cells and prevented the inhibitory effect of CEL on *T. cruzi* infection. Although not tested explicitly, we speculate that PGE_2_ inhibition by CEL leads to a reduction in intracellular cAMP (Saini et al., 2003), accounting for the anti-*T.cruzi* activities of CEL observed in this study. In according, we showed that treatment of DC-PBMCs with CEL but not with ASA in a combination with forskolin (activator of adenylyl cyclase) restored the infectivity of trypomastigotes for DC-PBMCs.

It has been described that *T. cruzi* can modulate cytokines and co-stimulatory molecules expression in DCs suggesting altered functions of these cells during infection, which might contribute controversially to both parasite immune evasion and an increased immune activation (Van Overtvelt et al., 1999; Gil-Jaramillo et al., 2016). Our observation that *T. cruzi* infection stimulated the release of TNF-α, IL-1β, IL-6, IL-8, IL-10 (involved in the control of intracellular infection) are in contrast with previous report showing no increase in the basal production of cytokines by human DCs infected with *T. cruzi* (Van Overtvelt et al., 1999). However, this effect presents similarity to infection by other pathogens, which induce DCs activation (Henderson et al., 1997; Gorak et al., 1998; Yamamoto et al., 1998; Amorim et al., 2016).

A small family of type1 glycoinositolphospholipids (GIPLs) is abundant in *T. cruzi* cell surface. Such molecules seem to have immunoregulatory functions (Brodskyn et al., 2002; Medeiros et al., 2007), as the inhibition of co-stimulatory molecules HLA-DR, CD83, CD86,CD80, and CD40 expression on DC surface, representing an evasion strategy of *T. cruzi* (Gil-Jaramillo et al., 2016). Our results indicate that the treatment with ASA or CEL did not promote any up-regulation of co-stimulatory molecules (CD80 and CD86).

In summary, our data show that trypomastigotes internalized by DC-PBMCs are able to survive intracellularly during infection, leading to high levels of inflammatory response. Alterations in PGE_2_ and cAMP levels can profoundly influence the immune functions of human DCs and alter the course of *in vitro T. cruzi* infection. This study strengthens that COX-2 pathway plays a fundamental role in the process of *T. cruzi* invasion. So, a deeper understanding of the mechanism of action of NSAIDs may indicate new targets for the control of Cd.

## Author contributions

SL, LR, LY, SY, and PP contributed to the conception of the study, JB, PP, and SY designed the study. AM, SY, HS, and PP carried out experiments, AM, HS, JB, and SY analyzed the data. SY and PP wrote the main manuscript text, JB and PP prepared manuscript figures. All authors reviewed the manuscript, contributed to the discussion and approved the final version.

### Conflict of interest statement

The authors declare that the research was conducted in the absence of any commercial or financial relationships that could be construed as a potential conflict of interest.

## Supplementary material

Figure S1 Characterization of DC-PBMC by CD11c and CD14 Cell surface staining. **(A)** Representative example of isotypic control and CD11c/CD14 labeled cells. **(B)** Representative graphs showing the percentage of CD11c^+^/ CD14^+^ and CD11c^+^/ CD14^−^. Results are the mean and standard error (95%) of four experiments with three independent donors.

Figure S2 Treatment with aspirin (ASA) or celecoxib (CEL) does not induce the activation of DC-PBMC. Expression levels of activation markers (CD86 and CD80) on uninfected DC-PBMC and treated with ASA or CEL **(A)**. Representative graphs showing the mean and standard error (95%) of four experiments with three independent donors **(B)**. No statistically significant differences (one-way ANOVA with Tukey's post-test) were observed between any of the groups analyzed.

## References

[B1] Abdalla G. K. Faria G. E. Silva K. T. Castro E. C. Reis M. A. Michelin M. A. (2008). *Trypanosoma cruzi*: the role of PGE2 in immune response during the acute phase of experimental infection. Exp. Parasitol. 118, 514–521. 10.1016/j.exppara.2007.11.003 18163990

[B2] Amorim K. N. Chagas D. C. Sulczewski F. B. Boscardin S. B. (2016). Dendritic cells and their multiple roles during malaria infection. J. Immunol. Res. 2016:2926436. 10.1155/2016/2926436 27110574 PMC4823477

[B3] Bern C. (2015). Chagas' disease. N. Engl. J. Med. 373, 1882 10.1056/NEJMra1410150 26535522

[B4] Brodskyn C. Patricio J. Oliveira R. Lobo L. Arnholdt A. Mendonca-Previato L.. (2002). Glycoinositolphospholipids from *Trypanosoma cruzi* interfere with macrophages and dendritic cell responses. Infect. Immun. 70, 3736–3743. 10.1128/IAI.70.7.3736-3743.2002 12065516 PMC128086

[B5] Caler E. V. Morty R. E. Burleigh B. A. Andrews N. W. (2000). Dual role of signaling pathways leading to Ca^(2+)^ and cyclic AMP elevation in host cell invasion by *Trypanosoma cruzi*. Infect. Immun. 68, 6602–6610. 10.1128/IAI.68.12.6602-6610.2000 11083771 PMC97756

[B6] Celentano A. M. Gorelik G. Solana M. E. Sterin-Borda L. Borda E. Gonzalez Cappa S. M. (1995). PGE2 involvement in experimental infection with *Trypanosoma cruzi* subpopulations. Prostaglandins 49, 141–153. 10.1016/0090-6980(95)00002-R 7652183

[B7] Coura J. R. Vinas P. A. (2010). Chagas disease: a new worldwide challenge. Nature 465, S6–S7. 10.1038/nature09221 20571554

[B8] de Araujo-Jorge T. C. (1989). The biology of *Trypanosoma cruzi*-macrophage interaction. Mem. Inst. Oswaldo Cruz 84, 441–462. 10.1590/S0074-02761989000400001 2487443

[B9] Dias J. C. Ramos A. N. Jr. Gontijo E. D. Luquetti A. Shikanai-Yasuda M. A. Coura J. R. (2016). 2 nd Brazilian consensus on Chagas disease, 2015. Rev. Soc. Bras Med. Trop. 49(Suppl. 1), 3–60. 10.1590/0037-8682-0505-2016 27982292

[B10] DosReis G. A. Lopes M. F. (2009). The importance of apoptosis for immune regulation in Chagas disease. Mem. Inst. Oswaldo Cruz 104(Suppl 1), 259–262. 10.1590/S0074-02762009000900033 19753482

[B11] Durand J. L. Mukherjee S. Commodari F. De Souza A. P. Zhao D. Machado F. S.. (2009). Role of NO synthase in the development of Trypanosoma cruzi-induced cardiomyopathy in mice. Am. J. Trop. Med. Hyg. 80, 782–787. 10.4269/ajtmh.2009.80.782 19407124 PMC2699411

[B12] Freire-de-Lima C. G. Nascimento D. O. Soares M. B. Bozza P. T. Castro-Faria-Neto H. C. de Mello F. G.. (2000). Uptake of apoptotic cells drives the growth of a pathogenic trypanosome in macrophages. Nature 403, 199–203. 10.1038/35003208 10646605

[B13] Ganzinelli S. Borda E. Joensen L. Sterin-Borda L. (2009). Chagasic antibodies induce cardiac COX-2/iNOS mRNA expression with PGE2/NO production. Int. J. Cardiol. 134, 212–223. 10.1016/j.ijcard.2008.02.008 18579232

[B14] Gil-Jaramillo N. Motta F. N. Favali C. B. Bastos I. M. Santana J. M. (2016). Dendritic cells: a double-edged sword in immune responses during Chagas disease. Front. Microbiol. 7:1076. 10.3389/fmicb.2016.01076 27471496 PMC4943928

[B15] Gorak P. M. Engwerda C. R. Kaye P. M. (1998). Dendritic cells, but not macrophages, produce IL-12 immediately following *Leishmania donovani* infection. Eur. J. Immunol. 28, 687–695. 10.1002/(SICI)1521-4141(199802)28:02<687::AID-IMMU687>3.0.CO;2-N 9521079

[B16] Guerrero N. A. Camacho M. Vila L. Iniguez M. A. Chillon-Marinas C. Cuervo H. (2015). Cyclooxygenase-2 and Prostaglandin E2 signaling through prostaglandin receptor ep-2 favor the development of Myocarditis during acute *Trypanosoma cruzi* infection. PLoS Negl. Trop. Dis. 9:e0004025 10.1371/journal.pntd.0004025 26305786 PMC4549243

[B17] Harizi H. Grosset C. Gualde N. (2003). Prostaglandin E2 modulates dendritic cell function via EP2 and EP4 receptor subtypes. J. Leukoc. Biol. 73, 756–763. 10.1189/jlb.1002483 12773508

[B18] Henderson R. A. Watkins S. C. Flynn J. L. (1997). Activation of human dendritic cells following infection with *Mycobacterium tuberculosis*. J. Immunol. 159, 635–643. 9218578

[B19] Hideko Tatakihara V. L. Cecchini R. Borges C. L. Malvezi A. D. Graca-de Souza V. K. Yamada-Ogatta S. F.. (2008). Effects of cyclooxygenase inhibitors on parasite burden, anemia and oxidative stress in murine *Trypanosoma cruzi* infection. FEMS Immunol. Med. Microbiol. 52, 47–58. 10.1111/j.1574-695X.2007.00340.x 18031539

[B20] Kalinski P. Hilkens C. M. Wierenga E. A. Kapsenberg M. L. (1999). T-cell priming by type-1 and type-2 polarized dendritic cells: the concept of a third signal. Immunol. Today 20, 561–567. 10.1016/S0167-5699(99)01547-9 10562707

[B21] Lopes M. F. DosReis G. A. (2000). Experimental Chagas disease: phagocytosis of apoptotic lymphocytes deactivates macrophages and fuels parasite growth. Apoptosis 5, 221–224. 10.1023/A:1009648311490 11225843

[B22] Lopez-Munoz R. Faundez M. Klein S. Escanilla S. Torres G. Lee-Liu D.. (2010). Trypanosoma cruzi: *in vitro* effect of aspirin with nifurtimox and benznidazole. Exp. Parasitol. 124, 167–171. 10.1016/j.exppara.2009.09.005 19735656

[B23] Machado F. S. Dutra W. O. Esper L. Gollob K. J. Teixeira M. M. Factor S. M.. (2012). Current understanding of immunity to *Trypanosoma cruzi* infection and pathogenesis of Chagas disease. Semin. Immunopathol. 34, 753–770. 10.1007/s00281-012-0351-7 23076807 PMC3498515

[B24] Machado F. S. Martins G. A. Aliberti J. C. Mestriner F. L. Cunha F. Q. Silva J. S. (2000). *Trypanosoma cruzi*-infected cardiomyocytes produce chemokines and cytokines that trigger potent nitric oxide-dependent trypanocidal activity. Circulation 102, 3003–3008. 10.1161/01.CIR.102.24.3003 11113053

[B25] Machado F. S. Mukherjee S. Weiss L. M. Tanowitz H. B. Ashton A. W. (2011). Bioactive lipids in *Trypanosoma cruzi* infection. Adv. Parasitol. 76, 1–31. 10.1016/B978-0-12-385895-5.00001-3 21884885 PMC3564251

[B26] Malvezi A. D. da Silva R. V. Panis C. Yamauchi L. M. Lovo-Martins M. I. Zanluqui N. G.. (2014a). Aspirin modulates innate inflammatory response and inhibits the entry of *Trypanosoma cruzi* in mouse peritoneal macrophages. Mediators Inflamm. 2014:580919. 10.1155/2014/580919 25045211 PMC4089847

[B27] Malvezi A. D. Panis C. da Silva R. V. Carvalho de Freitas R. Martins M. I. Tatakihara V. L. (2014b). Inhibition of cyclooxygenase-1 and cyclooxygenase-2 impairs *Trypanosoma cruzi* entry in cardiac cell and promotes differential modulation of inflammatory response. Antimicrob. Agents Chemother. 58, 6157–6164. 10.1128/AAC.02752-14 25092706 PMC4187892

[B28] Maya J. D. Orellana M. Ferreira J. Kemmerling U. Lopez-Munoz R. Morello A. (2010). Chagas disease: present status of pathogenic mechanisms and chemotherapy. Biol. Res. 43, 323–331. 10.4067/S0716-97602010000300009 21249304

[B29] Medeiros M. M. Peixoto J. R. Oliveira A. C. Cardilo-Reis L. Koatz V. L. Van Kaer L.. (2007). Toll-like receptor 4 (TLR4)-dependent proinflammatory and immunomodulatory properties of the glycoinositolphospholipid (GIPL) from *Trypanosoma cruzi*. J. Leukoc. Biol. 82, 488–496. 10.1189/jlb.0706478 17540734

[B30] Molina-Berrios A. Campos-Estrada C. Henriquez N. Faundez M. Torres G. Castillo C. (2013). Protective role of acetylsalicylic acid in experimental *Trypanosoma cruzi* infection: evidence of a 15-epi-lipoxin A(4)-mediated effect. PLoS Negl. Trop. Dis. 7:e2173 10.1371/journal.pntd.0002173 23638194 PMC3630130

[B31] Moll H. Flohe S. Rollinghoff M. (1995). Dendritic cells in *Leishmania major*-immune mice harbor persistent parasites and mediate an antigen-specific T cell immune response. Eur. J. Immunol. 25, 693–699. 10.1002/eji.1830250310 7705398

[B32] Moraes K. C. Diniz L. F. Bahia M. T. (2015). Role of cyclooxygenase-2 in *Trypanosoma cruzi* survival in the early stages of parasite host-cell interaction. Mem. Inst. Oswaldo Cruz 110, 181–191. 10.1590/0074-02760140311 25946241 PMC4489448

[B33] Mukherjee S. Machado F. S. Huang H. Oz H. S. Jelicks L. A. Prado C. M.. (2011). Aspirin treatment of mice infected with *Trypanosoma cruzi* and implications for the pathogenesis of Chagas disease. PLoS ONE 6:e16959. 10.1371/journal.pone.0016959 21347238 PMC3039660

[B34] Palucka K. Banchereau J. (2002). How dendritic cells and microbes interact to elicit or subvert protective immune responses. Curr. Opin. Immunol. 14, 420–431. 10.1016/S0952-7915(02)00365-5 12088675

[B35] Pinge-Filho P. Tadokoro C. E. Abrahamsohn I. A. (1999). Prostaglandins mediate suppression of lymphocyte proliferation and cytokine synthesis in acute *Trypanosoma cruzi* infection. Cell. Immunol. 193, 90–98. 10.1006/cimm.1999.1463 10202116

[B36] Procopio D. O. Teixeira M. M. Camargo M. M. Travassos L. R. Ferguson M. A. Almeida I. C.. (1999). Differential inhibitory mechanism of cyclic AMP on TNF-α and IL-12 synthesis by macrophages exposed to microbial stimuli. Br. J. Pharmacol. 127, 1195–1205. 10.1038/sj.bjp.0702624 10455266 PMC1566108

[B37] Rodriguez A. Martinez I. Chung A. Berlot C. H. Andrews N. W. (1999). cAMP regulates Ca^2+^-dependent exocytosis of lysosomes and lysosome-mediated cell invasion by trypanosomes. J. Biol. Chem. 274, 16754–16759. 10.1074/jbc.274.24.16754 10358016

[B38] Rodriguez P. Carlier Y. Truyens C. (2012a). Activation of cord blood myeloid dendritic cells by *Trypanosoma cruzi* and parasite-specific antibodies, proliferation of CD8+ T cells, and production of IFN-gamma. Med. Microbiol. Immunol. 201, 157–169. 10.1007/s00430-011-0217-y 22037700

[B39] Rodriguez P. Carlier Y. Truyens C. (2012b). *Trypanosoma cruzi* activates cord blood myeloid dendritic cells independently of cell infection. Med. Microbiol. Immunol. 201, 287–296. 10.1007/s00430-012-0230-9 22327272

[B40] Saini S. S. Gessell-Lee D. L. Peterson J. W. (2003). The cox-2-specific inhibitor celecoxib inhibits adenylyl cyclase. Inflammation 27, 79–88. 10.1023/A:1023226616526 12797547

[B41] Sallusto F. Lanzavecchia A. (1994). Efficient presentation of soluble antigen by cultured human dendritic cells is maintained by granulocyte/macrophage colony-stimulating factor plus interleukin 4 and downregulated by tumor necrosis factor alpha. J. Exp. Med. 179, 1109–1118. 10.1084/jem.179.4.1109 8145033 PMC2191432

[B42] Silva J. S. Machado F. S. Martins G. A. (2003). The role of nitric oxide in the pathogenesis of Chagas disease. Front. Biosci. 8, s314–s325. 10.2741/1012 12877141

[B43] Sterin-Borda L. Gorelik G. Goren N. Cappa S. G. Celentano A. M. Borda E. (1996). Lymphocyte muscarinic cholinergic activity and PGE2 involvement in experimental *Trypanosoma cruzi* infection. Clin. Immunol. Immunopathol. 81, 122–128. 10.1006/clin.1996.0167 8906742

[B44] Van Overtvelt L. Vanderheyde N. Verhasselt V. Ismaili J. de Vos L. Goldman M.. (1999). *Trypanosoma cruzi* infects human dendritic cells and prevents their maturation: inhibition of cytokines, HLA-DR, and costimulatory molecules. Infect. Immun. 67, 4033–4040. 10417171 10.1128/iai.67.8.4033-4040.1999 PMC96695

[B45] Watanabe Costa R. da Silveira J. F. Bahia D. (2016). Interactions between *Trypanosoma cruzi* secreted proteins and host cell signaling pathways. Front. Microbiol. 7:388. 10.3389/fmicb.2016.00388 27065960 PMC4814445

[B46] Wilkinson S. R. Bot C. Kelly J. M. Hall B. S. (2011). Trypanocidal activity of nitroaromatic prodrugs: current treatments and future perspectives. Curr. Top. Med. Chem. 11, 2072–2084. 10.2174/156802611796575894 21619510

[B47] World Health Organization (2017). World Health Organization: Chagas Disease. Available online at: http://www.who.int/mediacentre/factsheets/fs340/en/

[B48] Yamamoto K. Akbar S. M. Masumoto T. Onji M. (1998). Increased nitric oxide (NO) production by antigen-presenting dendritic cells is responsible for low allogeneic mixed leucocyte reaction (MLR) in primary biliary cirrhosis (PBC). Clin. Exp. Immunol. 114, 94–101. 10.1046/j.1365-2249.1998.00696.x 9764609 PMC1905073

[B49] Zingales B. Andrade S. G. Briones M. R. Campbell D. A. Chiari E. Fernandes O.. (2009). A new consensus for *Trypanosoma cruzi* intraspecific nomenclature: second revision meeting recommends TcI to TcVI. Mem. Inst. Oswaldo Cruz 104, 1051–1054. 10.1590/S0074-02762009000700021 20027478

