# Supplementary figures and images for: Response to Trypanosoma cruzi by Human Blood Cells Enriched with Dentritic Cells Is Controlled by Cyclooxygenase-2 Pathway
Source: Front Microbiol. 2017 Oct 25;8:2020. doi: 10.3389/fmicb.2017.02020 (PMC5660982; doi:10.3389/fmicb.2017.02020)

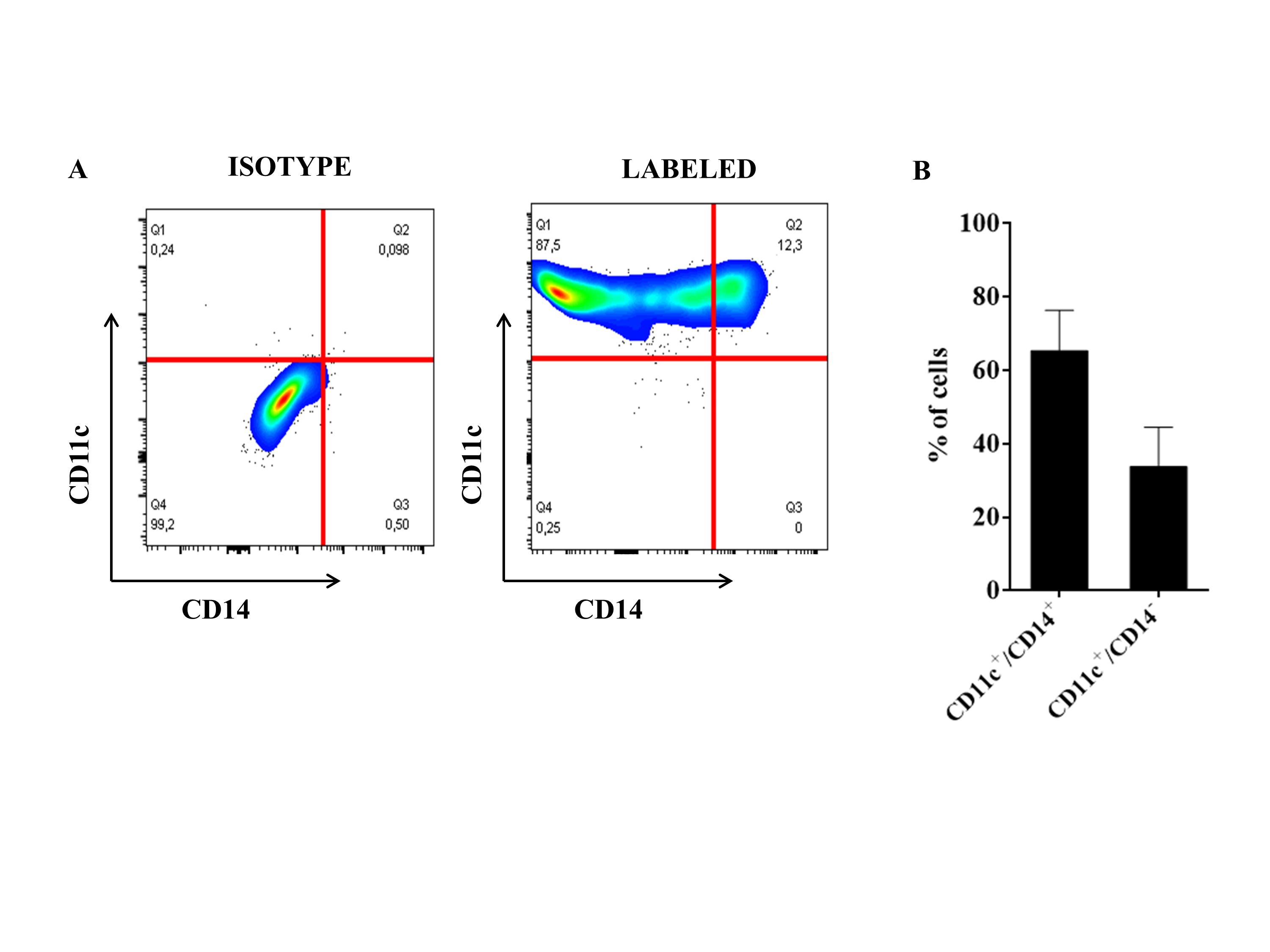

Supplement: Figure S1 — Characterization of DC-PBMC by CD11c and CD14 Cell surface staining. (A) Representative example of isotypic control and CD11c/CD14 labeled cells. (B) Representative graphs showing the percentage of CD11c+/ CD14+ and CD11c+/ CD14−. Results are the mean and standard error (95%) of four experiments with three independent donors. [file Image1.TIF]

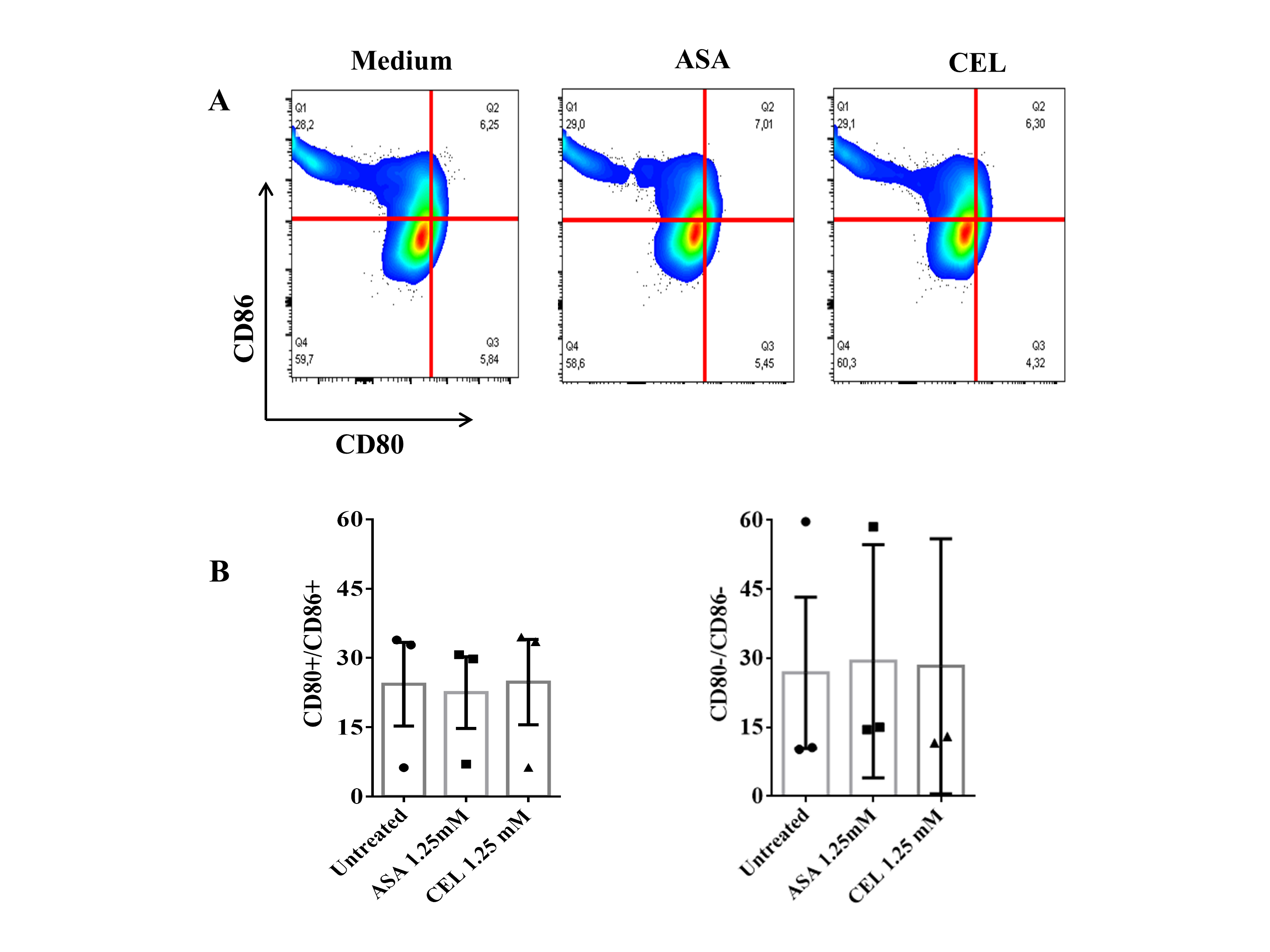

Supplement: Figure S2 — Treatment with aspirin (ASA) or celecoxib (CEL) does not induce the activation of DC-PBMC. Expression levels of activation markers (CD86 and CD80) on uninfected DC-PBMC and treated with ASA or CEL (A). Representative graphs showing the mean and standard error (95%) of four experiments with three independent donors (B). No statistically significant differences (one-way ANOVA with Tukey's post-test) were observed between any of the groups analyzed. [file Image2.TIF]
